# Indole-3-butyric acid promotes adventitious rooting in *Arabidopsis thaliana* thin cell layers by conversion into indole-3-acetic acid and stimulation of anthranilate synthase activity

**DOI:** 10.1186/s12870-017-1071-x

**Published:** 2017-07-11

**Authors:** L. Fattorini, A. Veloccia, F. Della Rovere, S. D’Angeli, G. Falasca, M. M. Altamura

**Affiliations:** Dipartimento di Biologia Ambientale, Sapienza Università di Roma, Roma, Italy

**Keywords:** Adventitious roots, Anthranilate synthase genes, *ech2ibr10* mutant, Indole-3-butyric acid, Indole-3-acetic acid, Indole-3-acetic acid influx carriers, Indole-3-acetic acid efflux carriers, In vitro culture, Nitric oxide, Stem thin cell layers

## Abstract

**Background:**

Indole-3-acetic acid (IAA), and its precursor indole-3-butyric acid (IBA), control adventitious root (AR) formation *in planta.* Adventitious roots are also crucial for propagation via cuttings. However, IBA role(s) is/are still far to be elucidated. In *Arabidopsis thaliana* stem cuttings, 10 μM IBA is more AR-inductive than 10 μM IAA, and, in thin cell layers (TCLs), IBA induces ARs when combined with 0.1 μM kinetin (Kin). It is unknown whether arabidopsis TCLs produce ARs under IBA alone (10 μM) or IAA alone (10 μM), and whether they contain endogenous IAA/IBA at culture onset, possibly interfering with the exogenous IBA/IAA input. Moreover, it is unknown whether an IBA-to-IAA conversion is active in TCLs, and positively affects AR formation, possibly through the activity of the nitric oxide (NO) deriving from the conversion process.

**Results:**

Revealed undetectable levels of both auxins at culture onset, showing that arabidopsis TCLs were optimal for investigating AR-formation under the total control of exogenous auxins. The AR-response of TCLs from various ecotypes, transgenic lines and knockout mutants was analyzed under different treatments. It was shown that ARs are better induced by IBA than IAA and IBA + Kin. IBA induced IAA-efflux (*PIN1*) and IAA-influx (*AUX1/LAX3*) genes, IAA-influx carriers activities, and expression of *ANTHRANILATE SYNTHASE -alpha1* (*ASA1*), a gene involved in IAA-biosynthesis. ASA1 and ANTHRANILATE SYNTHASE -beta1 (ASB1), the other subunit of the same enzyme, positively affected AR-formation in the presence of exogenous IBA, because the AR-response in the TCLs of their mutant *wei2wei7* was highly reduced. The AR-response of IBA-treated TCLs from *ech2ibr10* mutant, blocked into IBA-to-IAA-conversion, was also strongly reduced. Nitric oxide, an IAA downstream signal and a by-product of IBA-to-IAA conversion, was early detected in IAA- and IBA-treated TCLs, but at higher levels in the latter explants.

**Conclusions:**

Altogether, results showed that IBA induced AR-formation by conversion into IAA involving NO activity, and by a positive action on IAA-transport and ASA1/ASB1-mediated IAA-biosynthesis. Results are important for applications aimed to overcome rooting recalcitrance in species of economic value, but mainly for helping to understand IBA involvement in the natural process of adventitious rooting.

**Electronic supplementary material:**

The online version of this article (doi:10.1186/s12870-017-1071-x) contains supplementary material, which is available to authorized users.

## Background

Roots of higher plants can be classified into primary roots (PRs), developed from the root pole of the embryo, and post-embryonic lateral and adventitious roots, developed after seed germination. Lateral roots (LRs) are formed by the pericycle of the PR, whereas adventitious roots (ARs) are formed *in planta* by tissues of the PR in secondary vascular structure, and, mainly, by tissues of the aerial organs [1]. Moreover, the formation of ARs is crucial for vegetative propagation via cuttings, and in horticulture and forestry the formation of ARs allows for the cloning of superior genotypes and is an essential part of the breeding programs [2, 3]. In different types of explants and species, indole-3-acetic acid (IAA), and its natural precursor indole-3-butyric acid (IBA) [4], are the main inducers of ARs, when applied exogenously, alone or combined with other phytohormones, e.g. cytokinin and methyl jasmonate (MeJA) [5, 6]. In *Arabidopsis thaliana* dark-grown seedlings, IAA is the endogenous inducer of AR formation from the hypocotyl, with biosynthesis, signalling, and transport involved [7–9], however the exogenous application of IBA (10 μM), combined or not with a cytokinin [kinetin (Kin)], improves AR formation [7, 9]. It has been demonstrated a long time ago that IBA applied at 10 μM in combination with 0.1 μM Kin induces AR formation in tobacco and arabidopsis thin cell layers (TCLs) [10, 11]. By contrast, the role of IBA alone in inducing AR formation has not yet demonstrated in this culture system. However, IBA, at 10 μM, induces AR formation in arabidopsis stem cuttings, and better than IAA at the same concentration [12]. *Arabidopsis thaliana* TCLs consist of stem inflorescence tissues external to the vascular system, i.e., epidermis, cortical parenchyma, endodermis and, occasionally of one or two layers of fibers [10]. In stem cuttings including the vascular system, it has been hypothesized that the promotion of AR formation by exogenous IBA occurs by an interaction with the endogenous IAA content [12], whereas there is no information about the endogenous IBA and IAA content in the TCLs. The first aim of the research was to determine the endogenous levels of IBA and IAA at the onset of the culture in the arabidopsis TCLs to establish whether IBA (alone or combined with Kin), and IAA (alone), might control AR formation either by an interaction with the endogenous auxin pool or by a total exogenous control.

*In planta*, IBA is an important component of the auxin pool [13], and in arabidopsis, there is evidence that it is inactive during its cell-to-cell transit, becoming active, by conversion to IAA, in the target cells only [14]. Moreover, by the use of seedlings of the *ech2ibr10* mutant, blocked into IBA-to-IAA-conversion [15], it has been recently shown that the promotion of AR formation by exogenous IBA alone (10 μM) requires conversion into IAA and interaction with ethylene signalling [9]. However, the possibility that IBA can promote the AR process *in planta* also in a way different from its conversion to IAA has been also hypothesized [9]. Moreover, nitric oxide (NO) is known to be an IAA downstream signal, but is known to derive from IBA-to-IAA conversion ([16], and references therein). NO positively affects AR formation in numerous explant types, e.g. cucumber hypocotyl explants [17], however, its role in adventitious rooting from TCLs is unknown.

Transcriptome analyses of tea cuttings and mung bean seedlings in response to IBA treatments show the existence of a lot of IBA-regulated genes associated with adventitious rooting, including genes coding for proteins involved in auxin signalling and cellular influx and efflux [18, 19]. In accordance, IAA transport via PIN-FORMED (PIN) efflux carriers, e.g. PIN1, and via influx carriers, i.e., AUXIN RESISTANT1 (AUX1) and LIKE AUXIN RESISTANT3 (LAX3), has been demonstrated to be essential for the AR process in arabidopsis seedlings treated without exogenous hormones and with IBA + Kin [7, 8, 20]. The same carriers are active in the IBA + Kin-cultured TCLs, and the AR response strongly declines in TCLs excised from the *aux1* and *lax3aux1* mutants [7, 8]. Moreover, *in planta*, the activity of the promoters of *PIN1* and *LAX3* increases in the wild type (WT), and the AR density decreases in the *lax3aux1* mutant, also in the presence of IBA alone (10 μM) [9]. This suggests that IBA is sufficient to stimulate IAA transport *in planta*, whereas it remains to be determined whether this occurs also in the TCLs.

The *WEAK ETHYLENE-INSENSITIVE2/ANTHRANILATE SYNTHASE alpha1* (*WEI2/ASA1*) and *WEI7/ANTHRANILATE SYNTHASE beta1* (*ASB1*) genes encode, respectively, the α- and β-subunits of anthranilate synthase, a rate-limiting enzyme of an early step of the tryptophan-dependent IAA biosynthesis [21]. In arabidopsis seedlings, by the use of the *wei2wei7* mutant and the *ASA1::GUS* and *ASB1::GUS* lines, it has been shown that the anthranilate synthase is required for AR formation*,* with the transcriptional induction of the α-anthranilate synthase isoform *(WEI2/ASA1)* mainly involved [9]. However, it is unknown whether the same genes are involved in the AR-formation in TCLs*.*

The second aim of the research was to understand whether IBA alone was able to induce AR formation in arabidopsis TCLs, in comparison with IBA + Kin, IAA alone and Kin alone, whether the IAA transport by PIN1, LAX3, and AUX1 was affected, whether an IBA conversion into IAA was needed and possibly involved NO formation, and whether an IAA biosynthesis by WEI2/ASA1 and WEI7/ASB1 was also involved.

Nitric oxide is known to activate genes involved in jasmonic acid (JA) biosynthesis [22]. In addition, methyl jasmonate (MeJA) is known to control IAA biosynthesis by enhancing both *ASA1* and *ASB1* expression [23]. In tobacco IBA + Kin-treated TCLs, when applied at 0.1 and 0.01 μM, MeJA is rapidly cleaved to JA, and JA action results into enhanced AR formation [6]. However, MeJA effects on *ASA1* expression in IBA-treated arabidopsis TCLs are presently unknown. It is important to underline that ARs are formed in the TCLs following the same developmental stages that characterize AR formation in entire hypocotyls [7]. This means that the study of AR formation in the TCLs is representative of the natural process occurring *in planta*. In addition, the strict and continuous interrelation between biosynthesis and utilization of IAA and its precursor IBA [13, 14] make it impossible to determine with certainty the role of IBA in the AR process *in planta*. By contrast, it may be determined in the TCLs. In fact, it has been reported that they are unable to produce ARs under hormone free (HF) conditions [10] as a possible consequence of a poor or no endogenous auxin(s) content.

Results show that endogenous IBA and IAA are undetectable at culture onset, and that the AR response is totally dependent on the exogenous auxin, IBA alone in particular. The expression and activities of AUX1 and LAX3 and the expression of *PIN1* occur in the IBA-alone-treated TCLs during AR formation, and the conversion of IBA into IAA, followed by NO formation, is strictly necessary. Exogenous IBA, either alone, or mainly when combined with MeJA, enhances the expression of *WEI2/ASA1*, and ASA1 and ASB1 positively affect AR-formation in the presence of exogenous IBA. Altogether the results demonstrate that the IBA-promotion of adventitious rooting in TCLs involves conversion into IAA and NO production, and promotes IAA biosynthesis and transport.

## Methods

### Plant material and growth conditions

Seeds of *Arabidopsis thaliana* Col, Col-0 and Col-gl1 ecotypes, of *ASA1::GUS, DR5::GUS, PIN1::GUS, AUX1::GUS,* and *LAX3::GUS* transgenic lines, and of *wei2-1wei7-1*, *lax3aux1-21* and *ech2-1ibr10-1* double mutants were stratified for 3 days at 4 °C under continuous darkness and sown on a commercial soil. The seeds of the *DR5::GUS* line and the *PIN1::GUS* line were a generous gift of Sabrina Sabatini (Sapienza University Rome) and Stefano Bencivenga (University of Milan), respectively. The seeds of the *AUX1::GUS* and *LAX3::GUS* lines and of the *lax3aux1-21* mutant were kindly provided by Malcom Bennett (University of Nottingham), and those of the *ech2-1ibr10-1* mutant by Bonnie Bartel (Rice University Huston). The seeds of the *wei2-1wei7-1* mutant and of the *ASA1::GUS* transgenic line were bought by NASC (Nottingham Arabidopsis Stock Centre, School of Biosciences, University of Nottingham, UK). The seeds of Col, Col-0 and Col-gl1 ecotypes came from stocks of our laboratory. The plantlets obtained from these seeds were grown until the reproductive stage (40 days after germination) in the same growth chamber, at 22 ± 2 °C, 70% humidity and long days (white light of 22 Wm^−2^ light intensity).

### TCL culture

Superficial TCLs, about 0.5 × 8 mm, composed by six cell layers including the epidermis, were excised from the internodes of the inflorescence stem. The TCLs were cultured, epidermal side up, under continuous darkness, at 22 ± 2 °C, up to day 15 on a medium consisting of MS [24] salts supplemented with 0.55 mM myo-inositol, 0.1 μM thiamine-HCL, 1% (*w*/*v*) sucrose, 0.8% agar (*w*/*v*) (pH 5.7) (HF medium). Col-0 TCLs were cultured on this medium with the addition of 10 μM IBA, 10 μM IBA plus 0.1 μM Kin, 0.1 μM Kin, 10 μM IAA, 10 μM IBA plus 0.01 μM MeJA, and under HF as experimental control. TCLs from the *ech2ibr10, wei2-1wei7-1,* and *lax3aux1-21* mutants and their WT were cultured with 10 μM IBA, 10 μM IAA or under HF. *ASA1::GUS* TCLs were cultured with either 10 μM IBA, or 10 μM IAA or IBA plus 0.01 μM MeJA. *DR5::GUS*, *PIN1::GUS, LAX3::GUS, AUX1::GUS* TCLs, and TCLs from Col and Col-gl1 ecotypes, were cultured with 10 μM IBA. One hundred explants per genotype and treatment were used per replicate. The pH was adjusted to 5.7 with 1 M NaOH before autoclaving. For macroscopic analyses, the explants of the WT and mutants were examined under a LEICA MZ8 stereomicroscope at culture end, and the AR response evaluated as the percentage of explants either remaining at the initial stage at culture end or forming macroscopic callus and ARs, and as mean number of ARs (±SE) per rooting explant.

### Histochemical analysis of GUS activity

TCLs of *DR5::GUS, PIN1::GUS, LAX3::GUS*, *AUX1::GUS, ASA1::GUS* lines were harvested at day 8 and day 15 of culture, and processed with the GUS staining as described by Willemsen et al. [25], with minor modifications, as reported by Veloccia et al. [9]. After infiltration for 15 min in a vacuum belljar, the samples were incubated at 37 °C in the dark either for 30 min (*DR5::GUS* and *LAX3::GUS*), or 45 min (*AUX1::GUS, ASA1::GUS*), or 2.5 h (*PIN1::GUS*). After GUS assay, the samples were fixed in 70% (*v*/v) ethanol, dehydrated by a graded ethanol series, embedded in Technovit 7100 (Heraeus Kulzer), longitudinally sectioned at 12 μm with a Microm HM 350 SV microtome (Microm, Walldorf, Germany), and observed under light microscopy.

### Hormone quantification

TCLs of Col-0 were collected at time 0 (i.e., soon after the excision) and conserved to −80 °C until the analyses. The extraction of IAA and IBA was performed using aliquots of 50 mg of TCLs according to Veloccia et al. [9]. Quantitative determinations of IAA and IBA were carried out by Rapid Resolution-Reversed Phase-HPLC (RR-RP-HPLC) separation followed by MS/MS detection with a triple quadrupole (QqQ) mass-spectrometer with an ESI-interface (G6420A Agilent Technologies, CA, USA). Pure standards, internal standards, and the quantification of the two auxins were according to Veloccia et al. [9].

### Nitric oxide detection

Intracellular NO content in Col-0 TCLs cultured with either 10 μM IBA or 10 μM IAA was quantified using the cell-permeable diacetate derivative diamino-fluorescein-FM (DAF-FMDA; Sigma) under epifluorescence microscopy. TCLs were incubated in 20 mM HEPES/NaOH buffer (pH 7.4) supplemented with 5 μM DAF-FMDA for 20 min [26] at 2, 3 and 6 days of culture, after having verified that no significant epifluorescence signal was detectable with the buffer alone (Additional file 1: Figure S1 a-b). After washing three times with the buffer to remove the excess of the fluorescent probe, TCLs were observed using a Leica DMRB microscope equipped with the specific set of filters (EX 450–490, DM 510, LP 515). The images were acquired with a LEICA DC500 digital camera and analysed with the IM1000 image-analysis software (Leica). Ten observations in each of 20 TCLs per treatment were randomly carried out, and the intensities of the fluorescence signal (in green colour) were quantified using the ImageJ software (National Institute of Health, Bellevue, WA, USA) and expressed in Arbitrary Units (AUs; from 0 to 255). The values were averaged and normalized to the control ones, i.e., to those measured in TCLs incubated in the buffer without the fluorescent probe.

### Statistical analysis

Data were expressed as means (±SE). One-way or two-way analysis of variance (ANOVA, *P* < 0.05) was used to compare effects of treatments and/or genotypes, and, if ANOVA showed significant effects, Tukey’s post-test was applied (GraphPad Prism 6.0). The significance of the differences between the percentages was statistically evaluated using χ^2^ test (*P* < 0.05). All the experiments were repeated three times in two following years, and similar results were obtained (data of the replicate from the second year shown).

## Results

### IBA induces AR formation in the TCLs independently of the addition of cytokinin, and better than IAA

TCLs of *Arabidopsis thaliana,* ecotype Col-0, were grown under darkness for 15 days in the presence of IBA alone (10 μM), Kin alone (0.1 μM), IBA (10 μM) + Kin (0.1 μM), IAA alone (10 μM), and HF (control). The aim was to evaluate whether AR formation was inducible by IBA without cytokinin, and in the affirmative case, whether IBA was more efficient than IAA.

**Fig. 1:**
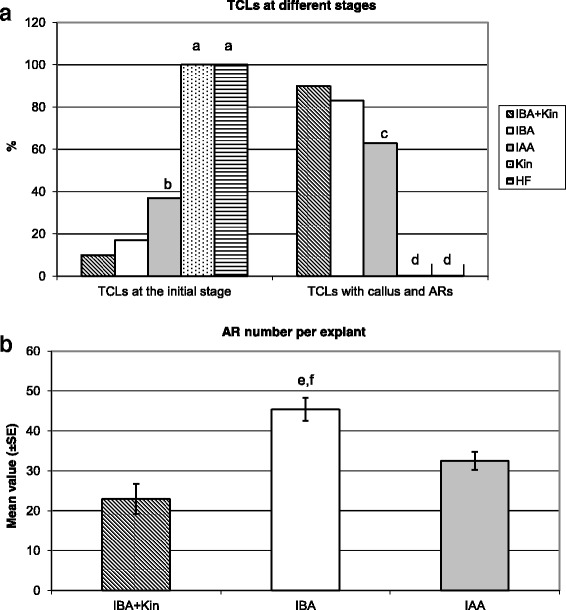
Adventitious rooting in *Arabidopsis thaliana* TCLs under different hormonal treatments. **a** Percentage of TCLs, Col-0 ecotype, either at the initial stage or with macroscopic callus and ARs, after 15 days of culture without hormones (HF) or with IBA (10 μM) + Kin (0.1 μM), IBA (10 μM), IAA (10 μM) or Kin (0.1 μM). **b** Productivity of AR-forming TCLs evaluated as mean number (±SE) of ARs per TCL under either IBA (10 μM) + Kin (0.1 μM), or IBA (10 μM), or IAA (10 μM). ^a,d^ *P* < 0.01 difference with respect to the other treatments within the same developmental stage. ^b,c^, *P* < 0.01 difference with respect to IBA + Kin and IBA within the same developmental stage. ^e^, *P* < 0.01 difference with respect to IAA. ^f^, *P* < 0.001 difference with respect to IBA + Kin. Columns with no letter or the same letter within the same developmental stage are not significantly different. *N* = 100

**Fig. 2:**
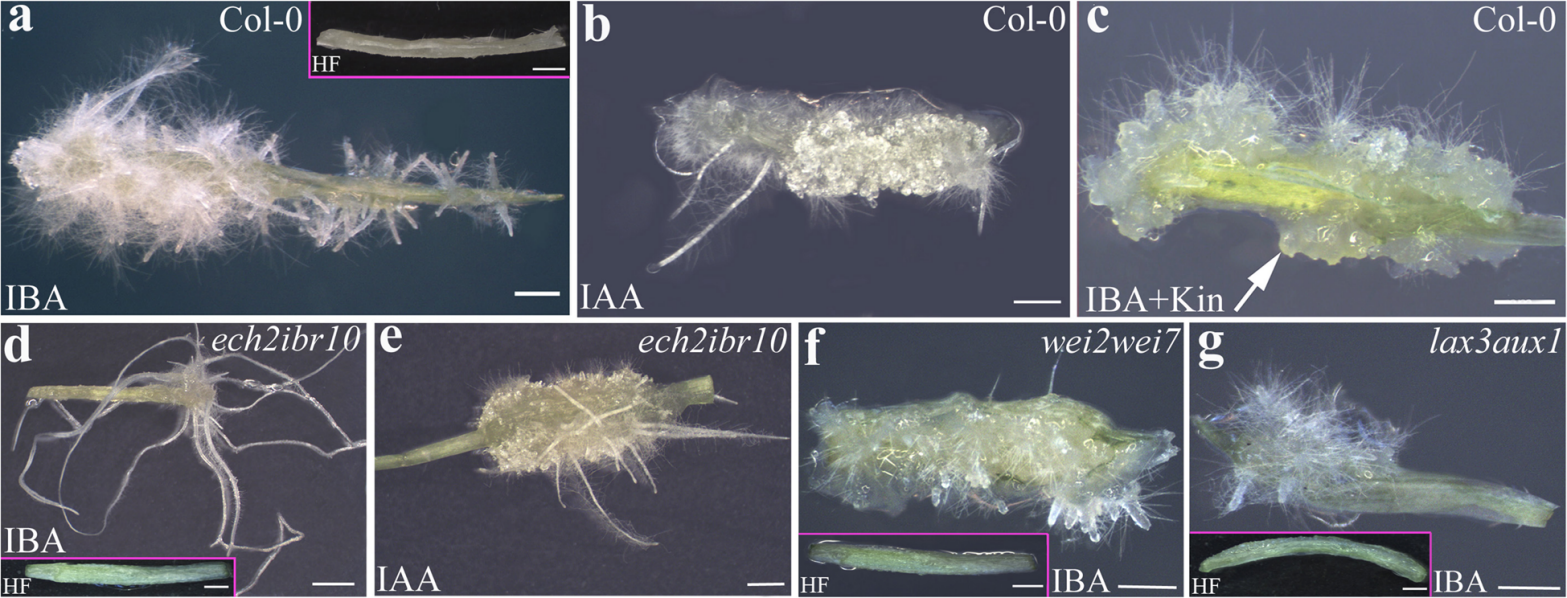
Macroscopic adventitious rooting response on TCLs from various genotypes under different hormonal treatments. **a**–**g** Images under the stereomicroscope at the end of the in vitro culture (day 15) with IBA (10 μM) (**a**, **d**, **f**, **g**), IBA (10 μM) + Kin (0.1 μM) (**c**), IAA (10 μM) (**b**, **e**) or under HF (Insets in **a**, **d**, **f**, **g**). **a** Col-0 TCLs showing a poor callus formation and a lot of elongated ARs with hairs. **b** Col-0 TCLs with elongated hairy ARs, and high callus formation. **c** Col-0 TCLs with ARPs (*arrow*) and no elongated AR, and callus. **d** *ech2ibr10* TCLs with a poor number of highly elongated ARs, and a very reduced callus formation. **e** *ech2ibr10* TCLs with callus and elongated ARs. **f**–**g** *wei2wei7* (**f**) and *lax3aux1* (**g**) TCLs with a very few number of ARs which were not elongated. Insets in **a**, **d**, **f**, and **g** show the absence of AR formation in the HF-treated control explants. Bars = 1 mm (**a**-**c**, **e**-**g,** and insets in **a**, **d**, **f**, **g**), 2 mm (**d**)

At culture end, the explants treated with Kin alone did not show any morphogenic/organogenic response, i.e., all remained at the initial stage, similarly to the explants cultured without hormones (Fig. 1a). By contrast, under the other three treatments the percentage of explants remaining at the initial stage at culture end was low, but it was significantly higher under IAA than under IBA + Kin and IBA alone (Fig. 1a). The percentage of explants with ARs followed an inverse trend, being very high under IBA + Kin (90%) and IBA (83%), without significant differences between the two, but under IAA it was significantly lower than IBA and IBA + Kin (Fig.1a). The AR production per explant was significantly higher under IBA alone than under IBA + Kin and IAA, whereas there was no significant difference between the latter two treatments (Fig. 1b). In addition, AR elongation and hair differentiation characterized both the IBA alone-treated explants and the IAA alone-treated ones, but the formation of calli was higher under IAA than IBA (Fig. 2a and b, in comparison). By contrast, ARs at the root primordium (ARP) stage were prevalently observed under IBA + Kin (Fig. 2c, arrow), and callus formation was higher than under IBA alone (Fig. 2a and c, in comparison). Interestingly, IBA alone treatment highly increased also the formation of LRs from the ARs in comparison with IBA + Kin (Additional file 1: Figure S1c-d). No HF-treated explant formed either ARs or macroscopic callus (Fig. 1a and Fig. 2a, inset).

To exclude possible differences in the AR response due to the genotype, the AR response of TCLs from different genotypes, i.e., Col-0, Col, and Col-gl1 was compared under IBA alone. No significant difference occurred in the mean number of ARs per explant (Additional file 1: Figure S1e). Thus, results from Figs. 1 and 2 show that 10 μM IBA was the best treatment for inducing AR formation in arabidopsis TCLs.

### Despite the absence of auxin at the excision time, IAA is active in the IBA-treated TCLs during AR formation

The steady state levels of endogenous IAA and IBA were measured in the TCLs soon after excision from the inflorescence stem. Neither IAA nor IBA were detected in the explants, showing that, in accordance with previous results in tobacco TCLs [6], the arabidopsis TCLs did not contain any endogenous auxin content at the excision time.

**Fig. 3:**
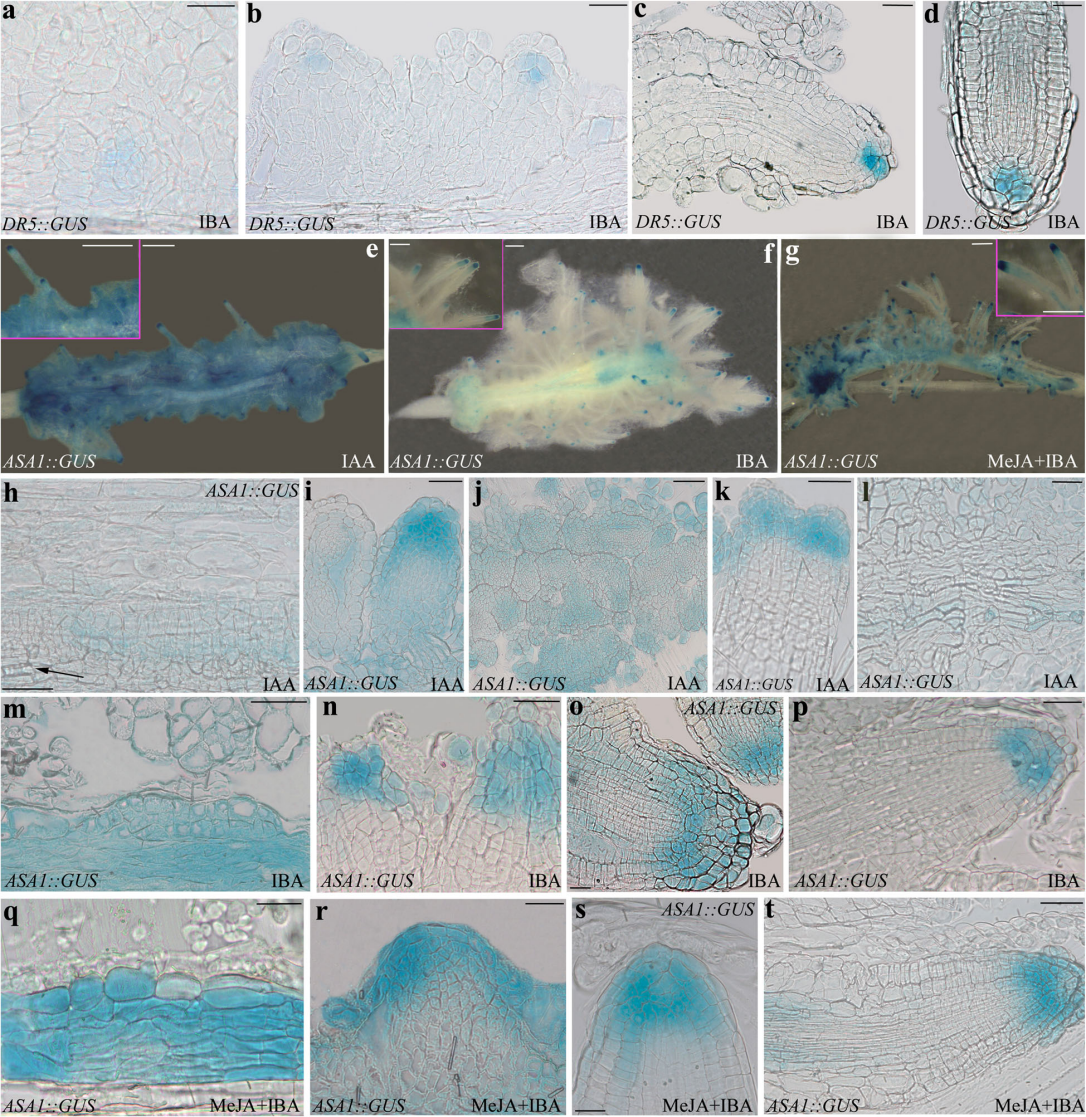
IAA-driven *DR5::GUS* expression in IBA-cultured TCLs, and *ASA1* expression under IAA, IBA, and MeJA + IBA. **a**–**d** Expression in TCLs treated with IBA (10 μM) for 8 (**a**-**b**) and 15 days (**c**-**d**). **a**-**b** Beginning of the signal in early meristemoids (**a**) and in ARPs (**b**). **c**-**d** *DR5*-signal in the quiescent centre and some initial and cap cells in the apex of elongating (**c**) and mature (**d**) ARs. **e**–**t** *ASA1::GUS* expression. **e**–**g** Expression in TCLs observed under the stereomicroscope at day 15 under IAA (10 μM) (**e**), IBA (10 μM) (**f**), or MeJA (0.01 μM) + IBA (10 μM) (**g**), showing differences in signal intensity and localization among the treatments at explant level, but not in the AR apex (Insets). **h**–**l** Histological analysis of the expression at 8 (**h**-**i**) and 15 (**j**-**l**) day in IAA-treated TCLs. **h**–**i** Expression in the endodermis derivatives (**h**) and in de novo formed xylary cells (*arrow*), and in the apex of the developing ARPs (**i**). **j**–**k** Widespread expression in the ARPs entrapped in the callus (**j**), and in the apices of the frequently fused ARs (**k**). **l** Faint expression in the meristematic cells of the xylogenic nodules. **m**–**p** Histological analysis of the expression at 8 (**m**-**n**) and 15 (**o**-**p**) days in IBA-treated TCLs. **m–n** High expression in the endodermis derivatives (**m**), and in the apical part of the forming ARPs (**n**). **o**–**p** Signal in the initial cells of the niche and in the protodermis of the apex of the elongating ARPs (**o**) and ARs (**p**), with faint expression in the columella in both cases. **q**–**t** Histological analysis of the expression at 8 (**q**-**r**) and 15 (**s**-**t**) days in MeJA + IBA-treated TCLs. **q**–**r** Strong signal in the endodermis derivatives (**q**), and in the apical part of the forming ARPs (**r**). **s**–**t** High signal in the initial cells of the niche and protodermis of the apex of the elongating ARPs (**s**), and ARs (**t**), with a lower expression in the columella in both cases. Bars = 20 μm (**r**, **s**), 40 μm (**a**-**c**, **h**, **i**, **k**-**p**, **q**, **t**), 50 μm (**d**, **j**, **o**), 500 μm (**e**-**g** and Insets)

The *DR5::GUS* line is a well-known reporter of the localization of IAA-induced gene expression in the AR-forming explants [7, 8, 27]. In entire portions of arabidopsis stem, the signal is occasional in the epidermal and cortical cells before the culture [8, 27]. By contrast, under the same plant growth and culture conditions presently used, in IBA + Kin TCLs the signal has been reported to appear in the meristematic cell clusters formed by the stem endodermis, and to continue in the meristemoids and tips of ARPs and ARs [7]. Because there is no information about the *DR5*-driven IAA signal in the presence of IBA alone in the TCLs, it was monitored histologically. A slight IAA-signal was observed in the meristematic cell clusters produced by the stem endodermis at day 8, and in the meristemoids (Fig. 3a) and ARPs (Fig. 3b). At day 15, the signal was reinforced in the tips of the elongating and mature ARs, marking the quiescent centre and some initial and cap cells around (Fig. 3c-d). Coupled with the initial absence of any endogenous IAA content in the TCLs, the results support that the IAA activity necessary for the AR process was totally dependent on the exogenous IBA input, sufficient per se to induce the IAA-signal specific for the AR process.

### The AR-response of *ech2ibr10* TCLs demonstrates that exogenous IBA is converted into IAA to induce AR formation

Mutations in genes encoding enzymes specific for IBA-to-IAA conversion confer IBA-resistance without altering IAA-response [28]. Among these enzymes, coded by genes with single alleles, there are the enoyl-CoA hydratase IBR10 and the ENOYL-COA HYDRATASE2 (ECH2). The *ibr10-1* mutant is resistant to the inhibitory effects of IBA on the elongation of light-grown roots [29], and dark-grown hypocotyls [15]. Moreover, *ibr10-1* produces dramatically fewer LRs than the WT in response to IBA [29]. Like *ibr10-1*, *eich2-1* mutant displays IBA-resistant hypocotyls and roots, and resistance is greatly enhanced when the two mutations are combined [15]. The synergism between the phenotypes of the two mutants is supported by the observation that, in comparison with the WT and the single mutants, *ech2ibr10* is unable to produce LRs in the absence of auxin exogenous treatments, and displays decreased auxin reporter activity [15]. About AR formation, it has been recently shown that *ech2ibr10* seedlings also show a reduced AR number in comparison with the WT in the presence of 10 μM IBA alone [9], whereas there is no information about *ech2ibr10* TCLs.

**Fig. 4:**
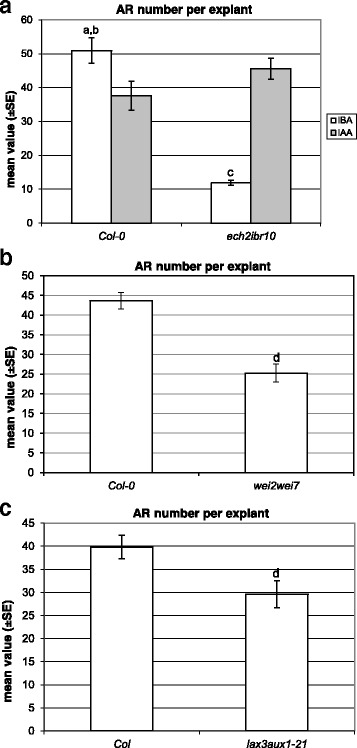
Adventitious rooting on TCLs from various genotypes cultured with IBA (10 μM) or IAA (10 μM). **a** Mean number (±SE) of ARs per IBA- and IAA-cultured TCL of Col-0 and *ech2ibr10* at day 15. **b** Mean number (±SE) of ARs per IBA-cultured TCL of Col-0 and *wei2wei7* at day 15. **c** Mean number (±SE) of ARs per IBA-cultured TCL of Col and *lax3aux1* at day 15. ^a^, *P* < 0.0001 difference with respect to *ech2ibr10* within the same treatment; ^b^, *P* < 0.05 and ^c^, *P* < 0.0001 difference with respect to IAA within the same genotype; ^d^, *P* < 0.01 difference with respect to the WT (Col-0 in **b**, Col in **c**). Columns with no letter are not significantly different. *N* = 100

In the absence of hormones, the *ech2ibr10* TCLs did not form ARs (Fig. 2d, inset), as the WT TCLs. The mean number of ARs per IBA-cultured TCL was about 5-folds lower than in the WT (*P* < 0.0001, Fig. 4a, and Fig. 2d and a, in comparison). This AR reduction was similar to that occurring in *ech2ibr10* seedlings grown under the same IBA concentration and experimental conditions [9]. Mutant and WT TCLs treated with IAA (Fig. 2e and b) showed the same high number of ARs (Fig. 4a). Taken together, data show that exogenous IBA needs to be converted into IAA to exhibit its action on AR formation in TCLs, as *in planta*.

### Both exogenous IAA and exogenous IBA induce the expression of the *ASA1* IAA-biosynthetic gene

To understand whether IBA had an effect on the IAA synthesis induced by the α subunit of the anthranilate synthase (WEI2/ASA1) [21], *ASA1* expression was evaluated in *ASA1::GUS* TCLs cultured with IBA alone, and the pattern compared with that obtained under IAA alone.

The expression pattern under either IAA alone or IBA alone showed that both the exogenous auxins were able to induce *ASA1* expression up to culture end (Fig. 3e-f), but the signal was extended to a wider portion of the callus under IAA in comparison with IBA (Fig. 3e-f), whereas the AR tips presented the same pattern of expression in both treatments (insets in Fig. 3e and f).

The histological analysis at day 8 of the IAA-treated TCLs showed that the expression started in the endodermis derivatives initiating both the AR process and the xylogenesis (Fig. 3h), and continued in the apical part of the forming ARPs (Fig. 3i). At day 15, the signal was diffused in the ARPs entrapped in the callus (Fig. 3j), but was also shown by the tips of the maturing ARs which were frequently fused (Fig. 3k). Moreover, the meristematic cells of the xylogenic nodules showed a faint expression (Fig. 3l).

In the presence of IBA, the expression pattern did not change in comparison with IAA alone (Fig. 3 m-p), however, at day 8, the signal in the endodermis derivatives was higher than with IAA alone (Fig. 3m and h, in comparison). In the elongating ARPs and in the ARs of day 15, the signal characterized the initial cells of the niche and the protodermis of the apical meristem, but faintly the columella (Fig. 3o-p). Xylogenesis sporadically occurred under IBA, but the forming xylary cells showed the same expression pattern as under IAA (data not shown).

### The induction of AR formation by exogenous IBA is reduced in the *wei2wei7* TCLs, supporting that ASA1/ASB activity is required for IBA-induced AR formation

For a deep insight into the action of exogenous IBA on AR formation from TCLs through the activity of WEI2/ASA1, but also of its isoform WEI7/ASB1, explants of the *wei2wei7* mutant, blocked at the level of both genes [21], were treated with 10 μM IBA, and the response compared with WT TCLs.

The mean number of ARs per IBA-treated TCL was significantly reduced in the double mutant in comparison with the WT (Fig. 4b and Fig. 2f and a, in comparison).

Coupled with the localization and timing of IBA-induced *ASA1* expression (Fig. 3m-p) and the *eich2ibr10* response (Fig. 4a), results demonstrate that exogenous IBA enhances AR formation in the TCLs through its conversion into IAA, with this event related to a stimulation of IAA biosynthesis by ASA1/ASB1.

### Exogenous IBA induces AUX1- and LAX3-mediated IAA-influx, and PIN1-mediated IAA-efflux in the AR-forming TCLs

The expression of *PIN1*, and of *AUX1* and *LAX3* genes, was analysed in the IBA-cultured TCLs.

**Fig. 5:**
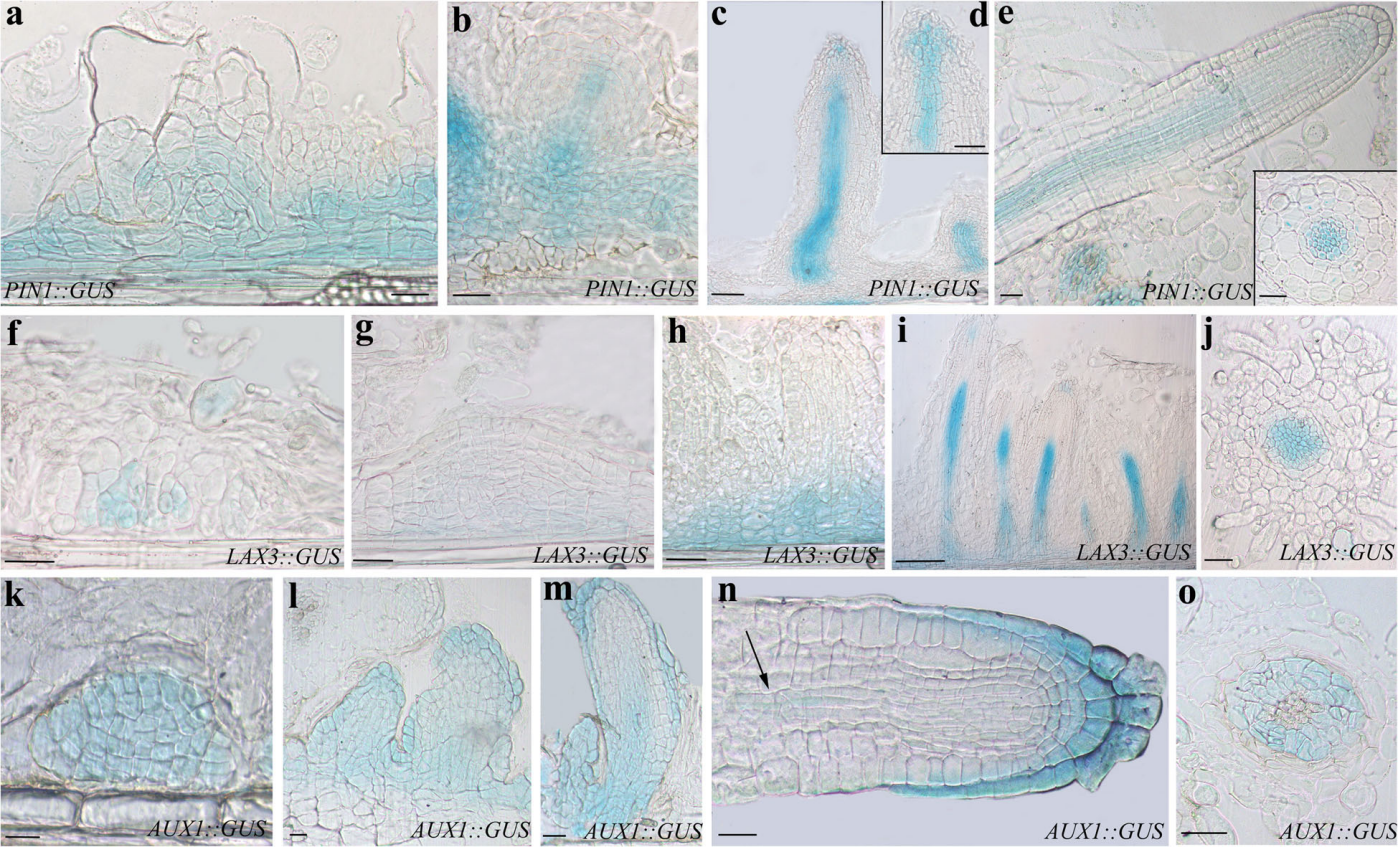
Expression pattern of *PIN1*, *LAX3*, and *AUX1* during AR formation in IBA-cultured TCLs at day15. **a**–**e** *PIN1::GUS* expression. **a** Signal in a wide population of endodermis derivatives and in meristemoids. **b**–**c** Signal in the basal part of young ARPs (**b)**, all along the developing vasculature (**c**), and in the central cells of the apex of elongating ARPs (**d**). **e** Expression in the vasculature (Inset), and faintly in the apex of mature ARs. **f**–**j** *LAX3::GUS* expression. **f**–**g** Onset of expression in the meristematic cell clusters formed by the endodermis. **h** Expression at the base of the differentiating ARPs (**h**). **i**–**j** Strong *LAX3* signal in the procambium (**i**), and vasculature of the maturing ARs (**j**). **k**–**o** *AUX1::GUS* expression. **k–l** Uniform signal in the meristematic cell clusters formed by the endodermis (**k**), and in early primordia (**l**). **m** Signal in the cap, protodermis, and developing procambium in the elongating ARPs. **n** *AUX1* expression in the cap, protodermis, and faintly in the niche and procambium (*arrow*) in a mature AR. **o** Expression in the vascular parenchyma of the AR primary structure zone. **a**-**e**, **f**-**i**, **k**-**n**, longitudinal sections, **j**, **o**, and Inset in **e**, transections. Bars = 40 μm (**a**, **b**, **f**–**k**, **n**, **o**), 50 μm (**c**-**e**, **l**, **m**, and Inset in **e**)

*PIN1* was expressed in a wide population of the endodermis derivatives (Fig. 5a), and at the base and along the procambium in the early ARPs (Fig. 5b). Moreover, in the elongating ARPs, *PIN1* was expressed in the differentiating vascular system and in the central part of the apical meristem (Fig. 5c and d). In the mature ARs, *PIN1::GUS* signal was present in the vasculature, and faintly at the apex (Fig. 5e and inset). Altogether the expression pattern of *PIN1* under IBA alone repeated that previously observed under IBA + Kin under experimentally comparable conditions [7], suggesting that IBA does not need Kin for causing IAA cellular efflux by PIN1 during AR formation in arabidopsis TCLs.

*LAX3* expression started in the meristematic cell clusters initiating the AR process (Fig. 5f-g), and continued at the base of the developing ARPs (Fig. 5h). In the elongated ARPs, the signal was present in the forming procambium and in a few apical cells (Fig. 5i). The expression signal remained in the vasculature of the maturing ARs (Fig. 5j). The expression pattern of *LAX3* did not differ from that previously observed under IBA + Kin with the same experimental conditions [7, 8].

The expression of *AUX1* started in the meristematic cell clusters, and was uniformly observed in all the cells of the early primordia and developing ARPs (Fig. 5k-l). The expression pattern changed in the elongated ARPs, because the signal was localized in the cap, protodermis, and developing procambium at ARP base (Fig. 5m). In the mature ARs, *AUX1* expression characterized the cap, the protodermis, and, faintly, some niche cells (Fig. 5n). The signal reappeared in the procambium of the differentiation zone (Fig. 5n, arrow), and, mainly, in the vascular parenchyma of the primary structure zone (Fig. 5o). Also in the case of *AUX1* there was no substantial difference in the expression pattern between the IBA-alone-treated TCLs and the previously examined IBA + Kin-cultured ones ([8], and present results).

To further support the importance of an IAA-influx mediated by the exogenous IBA in the target cells of the AR process, the response of the TCLs from *lax3aux1* double mutant was investigated, and compared with that occurring under IAA alone.

The IBA-treated TCLs of this double mutant showed an AR response significantly reduced in comparison with the WT both as percentage of AR-forming explants (40%, *P* < 0.01 difference with the WT) and as mean number of macroscopic ARs per explant (Figs. 2g and 4c).

The results support that exogenous IBA activates the IAA influx by AUX1 and LAX3 in the AR forming WT-TCLs. It cannot be excluded that the IBA-promotion of the activity of these IAA-transporters involved NO formation.

### Nitric oxide and methyl jasmonate enhance AR-formation in IBA-cultured TCLs

**Fig. 6:**
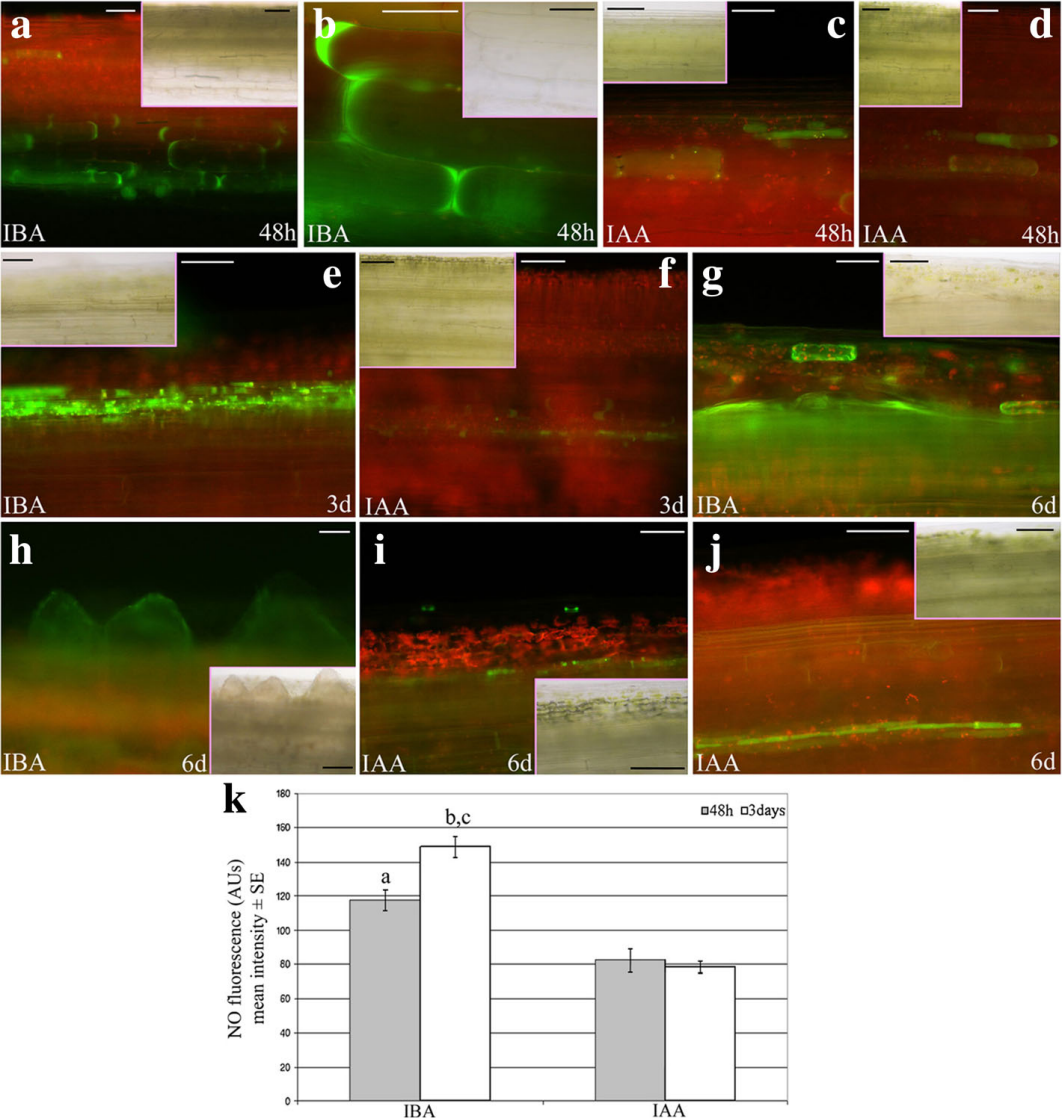
Detection and quantification of the epifluorescence signal caused by NO in IBA- or IAA-cultured TCLs. **a**–**d** Presence of the epifluorescence signal (*green colour*) at 48 h in cells of the deepest layers of TCLs cultured with IBA (10 μM) (**a**-**b**), or IAA (10 μM) (**c**-**d**). **e** Numerous endodermis derivative cells showing the NO green signal in TCLs cultured with IBA for 3 days. **f** Rare cells with a faint signal in the deepest layers of the explant in the presence of IAA at day 3. **g** Detail of the numerous layers of the endodermis derivatives showing the green epifluorescence at day 6 (IBA treatment). **h** Presence of the green signal in the first formed ARPs (day 6, IBA treatment). **i**–**j** Very faint signal in scattered cells (**i**), and in thin-layered endodermis derivatives (**j**) of the explant at day 6 (IAA treatment). TCL longitudinal views. The same images under light microscopy are shown in the Insets. **k** Mean intensity (±SE) of NO fluorescence (AUs) in TCLs cultured with either IBA (10 μM) or IAA (10 μM) for 48 h and 3 days. ^a,b^, *P* < 0.0001 difference with IAA within the same culture time. ^c^, *P* < 0.001 difference with the other culture time within the same treatment. Columns with no letter are not significantly different. *N* = 200. Bars = 50 μm (**b**, **c**, **e**, **g**–**j** and Insets in **b**, **f**, **g**, **i**), 70 μm (**a**, **d**, **f**, and Insets in **c**, **e**, **h**, **j**), 100 μm (Insets in **a** and **d**)

Nitric oxide is an IAA downstream signal, and an early by-product of IBA-to-IAA conversion [16]. Moreover, it is known to be positively involved in AR formation [17]. At 48 h, NO presence under IBA treatment was detected in a wider number of cells than under IAA (Fig. 6a-b and c-d, in comparison), but with a similar localization (cells of the deepest explant layers). At day 3, the difference of the epifluorescence signal became more evident between the auxin treatments. In fact, a lot of the endodermis derivatives showed the signal in the presence of IBA (Fig. 6e), whereas epifluorescence remained in scattered cells in the presence of IAA (Fig. 6f). Interestingly, at day 6, in the presence of exogenous IBA, entire layers of derivatives of the stem endodermis appeared green fluorescent, the same as the first formed ARPs (Fig. 6g-h). By contrast, only scattered cells or thin-layered endodermis derivatives were epifluorescent in the presence of exogenous IAA (Fig. 6i-j).

The intensity of green fluorescence was also quantified, and significant (*P* < 0.0001) increases occurred in the presence of IBA alone in comparison with IAA alone at both day 2 and day 3 of culture (Fig. 6k), supporting the microscopic observations.

Nitric oxide is known to activate genes of the JA biosynthesis [22], and MeJA treatments are known to enhance *ASA1* and *ASB1* expression [23].

To obtain information about NO downstream signals affecting AR formation in the TCLs, MeJA was applied at 0.01 μM [6] in combination with IBA (10 μM). At day 15, the treatment resulted into a significant (*P* < 0.01) increase in the mean number of ARs per TCL in comparison with IBA alone, i.e., 50 ± 2.5 and 40 ± 2.4, respectively. Moreover, *ASA1* expression signal increased in the TCLs under 0.01 μM MeJA + IBA in comparison with IBA alone (Fig. 3 f and g), without changing the expression pattern during the entire AR process (Fig. 3q-t and m-p, in comparison).

Altogether, results support a positive involvement of jasmonates, possibly formed downstream to NO, on ASA1/ASB1 expression/activity during IBA-mediated AR formation in TCLs.

## Discussion

Results showed that exogenous IBA alone induced AR-formation in arabidopsis TCLs. The AR-process was totally under the control of this exogenous auxin because the TCLs were devoid of IAA and IBA at culture onset. However, IBA needed to be converted into IAA to give AR formation, and favoured IAA-transport by PIN1, AUX1 and LAX3, and ASA1/ASB1-mediated IAA-biosynthesis. The latter two roles seemed to involve the action of the NO formed during the conversion process.

### The IAA-precursor IBA is the main player of the AR process positively affecting the biosynthesis of IAA, but its action is indirect

IBA is present in numerous plant species, in which it represents a variable percentage of the total pool of auxins. In arabidopsis seedlings, IBA levels are low, and differ depending on growth conditions and detection methodologies [30, 31]. However, IBA levels increase *in planta* when the AR process occurs. In fact, in arabidopsis AR forming hypocotyls of dark-grown seedlings, both IAA and IBA are present, with IBA levels about 10% of IAA levels. When exogenous IBA is applied at 10 μM, the endogenous IBA level triplicates, whereas endogenous IAA doubles [9]. This result shows that, in arabidopsis, as in other plants/culture systems, exogenous IBA is converted into endogenous IAA, and acts as source of IAA [12, 32, 33].

It has been suggested that the ASA1/ASB1-system functions when the auxin biosynthetic pathway is hyperactive [21]. In accordance, the endogenous IAA deficiency at the excision time presently observed in the TCLs might trigger a feedback loop, with exogenous IAA rapidly inducing its own biosynthesis via ASA1, as confirmed by the observed *ASA1::GUS* signal (Fig. 3h-l). This biosynthetic activity might cause an initial rise in the endogenous IAA, which might be useful for rapidly inducing both AR-formation and xylogenesis. In fact, also the latter program is auxin-inducible [34], and uses auxin to form xylary cells in competition with AR formation ([3], and references therein). The xylogenic response, frequently observed in the TCLs treated with IAA (Fig.3 h,l), but occasional in those treated with IBA, might explain the final reduction in AR formation occurring in the former treatment in comparison with the latter (Fig. 1).

By the use of the *DR5::GUS* system, it has been demonstrated that in arabidopsis stem cuttings exposed to IBA, the GUS signal appears, and is mainly associated with the root initiation sites [27]. Also in arabidopsis TCLs cultured with IBA + Kin for 14 days under the same experimental conditions presently used, the *GUS* signal characterizes the cells initiating the AR process, but is also observed in the de novo formed ARPs and ARs [7, 8]. Present results show that this pattern also occurs in the TCLs cultured with IBA alone, supporting that this exogenous auxin is able per se to induce IAA biosynthesis in the explants.

Recent transcriptome analyses of IBA-induced AR formation in *Camellia sinensis* cuttings and mung bean seedlings have allowed the identification of a lot of differentially expressed genes, and mainly genes involved in auxin homeostasis and signalling [18, 19]. However, no *ASA1* expression has been shown to be activated by IBA. By contrast, by the analysis of the expression pattern of *ASA1* under IBA alone (Fig. 3m-p), and the highly-reduced AR response of *wei2wei7* TCLs under the same treatment (Fig. 4b), it is presently shown that exogenous IBA induces *ASA1*, and the rooting promotion by IBA requires ASA1/ASB1. IBA conversion to IAA is catalysed by the action of peroxisomal ß-oxidation enzymes, e.g., IBR10 and ECH2 [15]. The highly-reduced AR-response observed in the *eich2ibr10* TCLs (Fig. 4 a) supports that the peroxisomal IBA-to-IAA conversion occurs in the IBA-treated TCLs. This conversion occurs also during AR formation in arabidopsis *in planta* [9], and during LR formation in arabidopsis and *Zea mays* [16]. In the latter study, it has been demonstrated that the conversion of IBA into IAA is followed by peroxisomal NO formation, and that the spatially and temporally coordinated release of NO and IAA from peroxisomes is the causative agent of the promotion of LR formation [16]. Nitric oxide also mediates the auxin response leading to AR formation [17, 35]. Not only IBA, but also IAA uses NO as downstream signal for LR formation, however, peroxisomes accumulate more NO under IBA treatment than under IAA ([16], and references therein). Present results show that this is also the case for AR formation from TCLs. In fact, an earlier and enhanced detection of NO occurred in the IBA-cultured explants in comparison with the IAA-cultured ones (Fig. 6a-d, and k), supporting an important involvement of the NO coming from IBA-to-IAA conversion. Taken together, NO might be a messenger in IBA-induced AR formation.

**Fig. 7:**
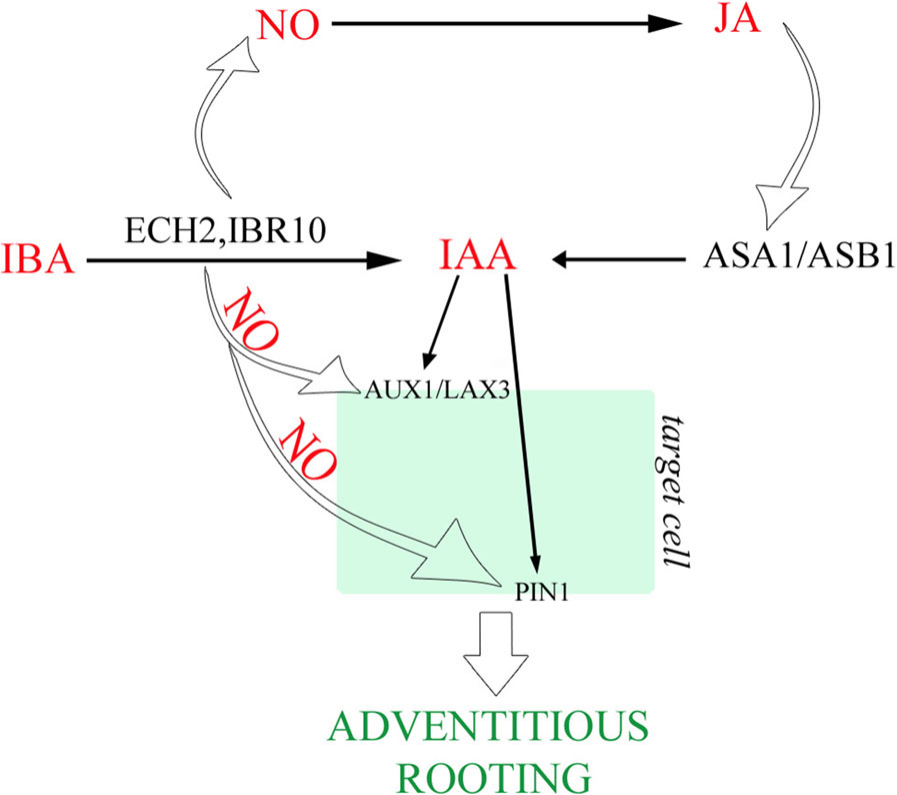
Model explaining the promotion by exogenous IBA (10 μM) of AR formation in arabidopsis TCLs. Nitric oxide (NO) formed during the exogenous IBA-to-IAA conversion by ECH2/IBR10 induces the synthesis of JA, which, in turn, induces ASA1/ASB1 activity. The IAA, coming from IBA conversion and biosynthesis by ASA1/ASB1, is transported into the target cells of the rhizogenic process by the efflux carrier PIN1 and the influx carriers AUX1 and LAX3. NO might also positively affect PIN1 and AUX1, enhancing the endogenous IAA transport required for adventitious rooting. (See the text for further explanations)

An interaction of NO with auxin synthesis and transport has been reported [36]. Moreover, NO is known to activate *Allene oxide synthase* (*AOS*) and *lypoxygenase2* (*LOX2*) genes involved in JA biosynthesis [22]. Jasmonates induce AR formation in arabidopsis (present results) and tobacco TCLs [6], when combined with exogenous IBA and IBA + Kin, respectively. ASA1 is required for the JA-induced IAA biosynthesis necessary to LR formation in arabidopsis [23]. Present results showed that MeJA, combined with IBA, enhanced *ASA1* expression in comparison with IBA alone without changing the expression pattern of the gene during the AR process (Fig. 3 q-t, and m-p, in comparison). Consequently, ASA1 might be an interaction node through which jasmonate integrates its action with auxin to regulate AR formation. In our hypothesis, the NO formed during the IBA-to-IAA peroxisomal conversion might induce *AOS* and *LOX2*, involved in JA biosynthesis, and the produced JA might induce ASA1/ASB1 expression/activity, increasing the IAA content coming from conversion, leading to the endogenous IAA pool necessary for AR formation (Fig. 7).

Only hypotheses may be advanced about the auxin signalling and perception necessary to successful AR formation in TCLs. It is known that genes that are up-regulated or down-regulated by auxin contain auxin response elements (AuxREs, 5′ tgtctc 3′) in their promoters, which bind transcription factors of the auxin response factor (ARF) family [37]. At high auxin levels, the ARFs become active because released by their repressors, the Aux/IAAs proteins, when the latter are degradated after interaction with the SCF^TIR1/AFB^ complex [38]. In intact hypocotyls of de-etiolated arabidopsis seedlings, AR initiation is controlled by a balance between the negative AR regulator ARF17 and the positive AR regulators ARF6 and ARF8, with these three ARFs controlling each other’s expression at both transcriptional and post-transcriptional level [39]. In contrast to ARF17, ARF6 and ARF8 positively affect the auxin-inducible genes GH3.3, GH3.5, and GH3.6, required for fine-tuning AR initiation by modulating JA homeostasis [40]. These results suggest a regulatory pathway at the crosstalk of IAA and JA, in which ARF6, ARF8, and ARF17 and their downstream targets GH3.3, GH3.5, and GH3.6 are involved. The same pathway might be active in the dark-grown AR-forming TCLs, with perhaps also NO involved. Nitric oxide might mediate auxin signalling via modification of the TIR1/AFB-Aux/IAA-ARF interaction. In fact, NO is known to enhance auxin signalling via S-nitrosylation of the auxin receptor protein TIR1, thereby facilitating Aux/IAA degradation [41]. Our preliminary results about the expression patterns of these *ARF* and *GH3* genes in dark-grown arabidopsis TCLs cultured with IBA + Kin support the hypothesis (Fattorini et al., unpublished results), which however needs to be confirmed by the response of the null mutants.

### The IAA-precursor IBA is the main player of the AR process positively affecting IAA transport, but its action is indirect

It has been suggested that AUX1 recognizes endogenous IAA and not IBA, whereas IBA may be a substrate of LAX3, at least in arabidopsis hypocotyls [42]. During AR formation in entire dark-grown seedlings, *AUX1* expression appears at the onset of the AR process, and continues during ARP formation, and in the ARs, but the pattern does not change under HF condition in comparison with IBA + Kin treatment, and remains the same also in IBA + Kin-treated TCLs [8]. *LAX3* expression is enhanced in the seedlings by IBA + Kin in comparison with the HF treatment, but also in this case there is no change in the IBA + Kin-induced pattern *in planta* in comparison with TCLs [7]. Present data support that the exogenous Kin does not affect the expression pattern of both these IAA-influx carriers in the arabidopsis TCLs, because IBA alone (Fig. 5f-o) did not induce any significant change in the expression pattern in comparison with previous results with IBA + Kin under comparable conditions [7, 8]. In tobacco TCLs [11], Kin had been supposed to act synergistically with IBA to induce the mitotic activity necessary for callus formation and meristemoid growth, and for this reason it had been then used for arabidopsis TCL culture [10]. By contrast, present results show that Kin is not necessary to arabidopsis TCLs, because the AR meristemoids are formed with IBA alone.

Moreover, present data also show a post-transcriptional role of exogenous IBA when applied alone, because the knockout of both *AUX1* and *LAX3* IAA-carrier genes caused a reduced AR response in comparison with the WT (Fig. 4c). Previous data show that TCLs from *aux1* mutant treated with IBA + Kin also have a reduced AR response in comparison with the WT, whereas this does not occur in *lax3* ones [8, 20], collectively suggesting a pivotal role for AUX1, independent/partially dependent on the exogenous hormonal treatment, in early controlling the AR process.

PIN proteins are encoded by a multigene family, with high homology among species. In *Medicago truncatula*, IAA treatments increase *MtPIN1* and *MtPIN2* expression, up-regulate most of the *PINs* in rice [43, 44], and positively affect *PIN1* promoter activity in arabidopsis PR [45]. Coupled with the inhibition of AR formation reported for *pin1* de-rooted seedlings [46], and the reduced AR response of *lax3/aux1* IBA-alone-treated seedlings, and IBA + Kin-treated TCLs [8, 9], the present results about *PIN1* and *AUX1* expression (Fig. 5a-e, and k-o) and *l*a*x3aux1* response (Fig. 4c) suggest that both AUX1 and PIN1 are activated by exogenous IBA in arabidopsis AR formation. However, IBA action would occur by the IAA coming from the IBA-to-IAA conversion. Following this hypothesis, in addition to the IAA formed by conversion, a by-product of the same conversion process, e.g., NO, would be another regulator of the action of AUX1 and PIN1. Post-translational modification, such as protein phosphorylation, is crucial for many aspects of functional biology of plant proteins, with NO as an important regulator ([47], and references therein). By a quantitative phosphoproteomics analysis of NO responsive phosphoproteins in cotton leaf, it has been recently demonstrated that both PIN1 and AUX1 are activated by NO-mediated phosphorylation [47]. Taken together, in the AR-forming IBA-cultured TCLs, the NO formed during the IBA-to-IAA conversion might not only affect the jasmonate-induced ASA1 expression/activity, but also enhance the endogenous IAA transport by phophorylation of both PIN1 and AUX1 (Fig. 7).

## Conclusions

IBA is the main player of adventitious rooting in arabidopsis TCLs, and possibly in many other culture systems and species characterized by very low endogenous auxin contents. IBA acts by conversion into IAA, and by enhancing IAA biosynthesis and transport. The nodal point of its action is the regulation of the endogenous IAA pool. IBA-regulation of IAA homeostasis involves the activity of other compounds downstream to its peroxisomal conversion, NO and jasmonates. The relationship of IBA with NO and jasmonates, and the downstream auxin signalling and perception, needs further investigation. Even if useful for planning experiments to overcome the rooting recalcitrance of species of economic value, the main implication of the findings is to help in understanding the mechanism by which IBA controls the natural process of adventitious rooting,

## Additional file

Additional file 1: Figure S1. NO-buffer controls, lateral-rooting on ARs of IBA- and IBA + Kin-cultured TCLs, and AR-formation on IBA-treated TCLs from different ecotypes. Description of data: (**a**-**b**) Autofluorescence signal in the explants after buffer (HEPES/NaOH) alone treatment at 48 h of culture and at day 3, under IBA (10 μM). The same images under light microscopy are shown in the Insets. (**c**) Percentage of ARs with lateral root (LR) formation on *Arabidopsis thaliana* TCLs, Col-0 ecotype, at the end (day 15) of culture on IBA (10 μM) + Kin (0.1 μM) or IBA (10 μM) alone. ^a^, *P* < 0.01 difference with IBA + Kin, *N* = 260. (**d**) Detail of a TCL from Col-0 ecotype at day 15 of culture on IBA, showing an AR with two LRs (*arrows*). (**e**) Mean number (±SE) of ARs per TCL from Col-0, Col, and Col-gl1 ecotypes after 15 days of culture with IBA (10 μM). *N* = 100. Columns with no letter are not significantly different. Longitudinal views (**a**-**b**), and stereomicroscope image (**d**). Bars = 20 μm (**a** and Inset in **a**), 40 μm (**b** and Inset in **b**), 200 μm (**d**). (PDF 3242 kb)

## Abbreviations

AOS: Allene Oxide Synthase
AR: Adventitious root
ARF: Auxin response factor
ASA1: ANTHRANILATE SYNTHASE-alpha1
ASB: ANTHRANILATE SYNTHASE-beta1
AUX1: AUXIN RESISTANT1
*ECH2*: *ENOYL*-*COA* *HYDRATASE2*
HF: Hormone free
IAA: Indole-3-acetic acid
IBA: Indole-3-butyric acid
*IBR10*: *INDOLE-3-BUTYRIC ACID RESPONSE10*
JA: Jasmonic acid
Kin: Kinetin
LAX3: LIKE AUXIN RESISTANT3
LOX2: Lypoxygenase 2
LR: Lateral root
MeJA: Methyl jasmonate
NO: Nitric oxide
PIN1: PIN-FORMED1
PR: Primary root
TCLs: Thin cell layers
WEI2: WEAK ETHYLENE-INSENSITIVE2
WEI7: WEAK ETHYLENE-INSENSITIVE7
WT: Wild type
