# Supplementary figures and images for: Indole-3-butyric acid promotes adventitious rooting in Arabidopsis thaliana thin cell layers by conversion into indole-3-acetic acid and stimulation of anthranilate synthase activity
Source: BMC Plant Biol. 2017 Jul 11;17:121. doi: 10.1186/s12870-017-1071-x (PMC5504571; doi:10.1186/s12870-017-1071-x)

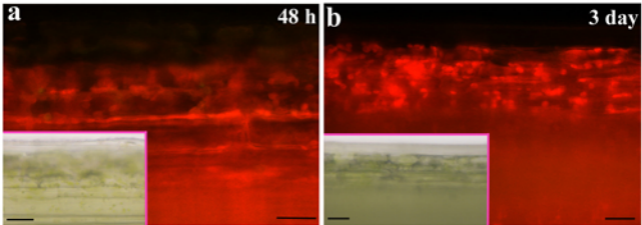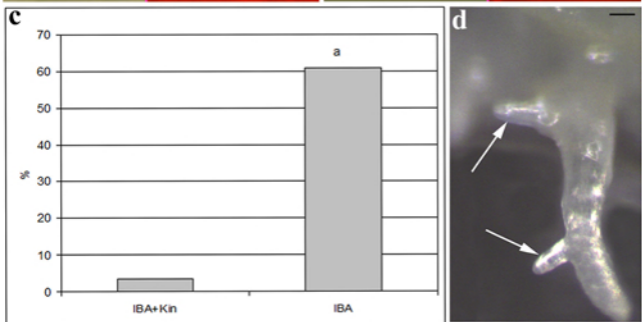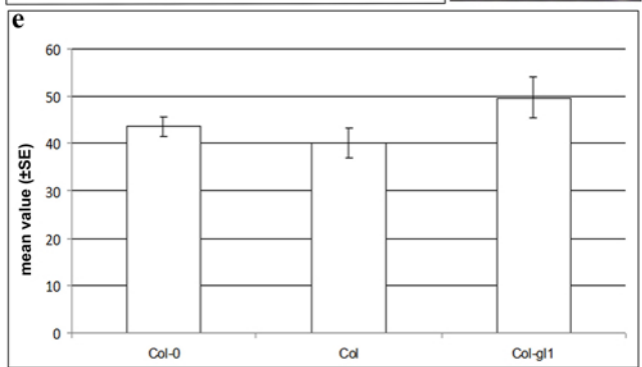

Supplement: Additional file 1: Figure S1. — NO-buffer controls, lateral-rooting on ARs of IBA- and IBA + Kin-cultured TCLs, and AR-formation on IBA-treated TCLs from different ecotypes. Description of data: (a-b) Autofluorescence signal in the explants after buffer (HEPES/NaOH) alone treatment at 48 h of culture and at day 3, under IBA (10 μM). The same images under light microscopy are shown in the Insets. (c) Percentage of ARs with lateral root (LR) formation on Arabidopsis thaliana TCLs, Col-0 ecotype, at the end (day 15) of culture on IBA (10 μM) + Kin (0.1 μM) or IBA (10 μM) alone. a, P < 0.01 difference with IBA + Kin, N = 260. (d) Detail of a TCL from Col-0 ecotype at day 15 of culture on IBA, showing an AR with two LRs (arrows). (e) Mean number (±SE) of ARs per TCL from Col-0, Col, and Col-gl1 ecotypes after 15 days of culture with IBA (10 μM). N = 100. Columns with no letter are not significantly different. Longitudinal views (a-b), and stereomicroscope image (d). Bars = 20 μm (a and Inset in a), 40 μm (b and Inset in b), 200 μm (d). (PDF 3242 kb) [file 12870_2017_1071_MOESM1_ESM.pdf]
